# Randomized Phase 1 Studies Evaluating the Safety, Tolerability, Pharmacokinetics, and Target Occupancy of Zampilimab in Healthy Participants

**DOI:** 10.1002/cpdd.70052

**Published:** 2026-05-13

**Authors:** Jo Collier, Rowann Bowcutt, Geoffrey I. Johnston, Jane Y. C. Chan, Anastasiia Raievska, Alison Bigley, Richard Nicholl, Tim S. Schmidt, Maria Sarno, Zahid Ali, Elizabeth Thomson

**Affiliations:** ^1^ UCB Slough UK; ^2^ Veramed London UK; ^3^ OracleBio Biocity Scotland UK; ^4^ Present address: AviadoBio London UK; ^5^ Present address: F. Hoffmann‐La Roche Basel Switzerland; ^6^ Present address: Senisca Ltd. Exeter UK; ^7^ Present address: Elizabeth Thomson Limited London UK

**Keywords:** drug safety, fibrosis, first‐in‐human Phase 1 study, nephrology, pharmacoepidemiology, TG2, target occupancy, zampilimab

## Abstract

Zampilimab (UCB7858) is a humanized monoclonal immunoglobulin G4P, transglutaminase 2 (TG2) inhibiting antibody. We investigated safety, tolerability, pharmacokinetics, and target occupancy of zampilimab (intravenous ≤2000 mg; subcutaneous ≤1000 mg) in healthy participants in a randomized, placebo‑controlled, single‐ascending‐dose Phase 1 study (UP0029; NCT02879877). Of 78 participants (58 zampilimab; 20 placebo), treatment‐emergent adverse events (TEAEs) occurred in 42 (72.4%) versus 14 (70%), respectively. One participant (intravenous zampilimab 2000 mg) reported a serious TEAE of moderate infusion‐related reaction (a protocol‐defined stopping criterion), leading to a temporary study hold. Although the reaction resolved within 3 days, and the participant completed follow up, dose‐escalation was suspended, and the study terminated. Zampilimab demonstrated dose‐proportional, linear pharmacokinetics (t_1/2_ 17‐23 days; subcutaneous bioavailability 77%). TG2 target occupancy in skin directly correlated with serum zampilimab concentration, near‐maximal occupancy ≥250 µg/mL, corresponding to intravenous doses ≥1000 mg between Days 2 and 5. A subsequent randomized, placebo‑controlled, Phase 1 study (UP0105; NCT04705350) investigated single intravenous doses of zampilimab (2000 and 3000 mg) at a lower concentration and slower infusion rate versus UP0029, using learnings from the administration strategy. Of 16 participants (12 zampilimab; four placebo), TEAEs occurred in eight (66.7%) versus one (25%), respectively. TEAEs were mild/moderate; none were drug related. No infusion‐related reactions occurred. Zampilimab safety profile was acceptable after single intravenous and subcutaneous doses ≤1000 mg (UP0029) and intravenous doses ≤3000 mg (UP0105) following optimization of maximum concentration (10 mg/mL) at an infusion rate of 25 mg/min over 120 min. These studies support the ongoing development of zampilimab.

Transglutaminases (TGs) are a family of catalytically‐active enzymes that post‐translationally modify proteins via protein cross‐linking, de‐amidation, or amine incorporation.^1^, ^2^ In addition, TGs act as scaffolds, help to maintain the structural integrity of membranes, regulate cell adhesion, and modulate signal transduction.^3^ TG2 is widely distributed in cells and tissues but is one of only two TGs released into the extracellular matrix (ECM), where its crosslinking role enhances matrix stability and contributes to wound healing.^3^, ^4^ Increased synthesis and extracellular trafficking of TG2 are associated with fibrosis and tissue scarring^5^, ^6^ in various organs, including the kidney,^6^, ^7^, ^8^, ^9^, ^10^, ^11^ lung,^12^, ^13^, ^14^, ^15^, ^16^ heart,^17^, ^18^ and liver,^19^, ^20^, ^21^ making this mechanism an ideal candidate as a therapeutic intervention for patients with fibrotic disease.^22^, ^23^

Zampilimab (UCB7858) is a humanized monoclonal immunoglobulin G4P (IgG4P) antibody that specifically blocks the enzymatic function of TG2^24^ and affects the development of fibrosis by directly interfering with ECM assembly, stabilization, and turnover.^24^, ^25^, ^26^ Zampilimab targets extracellular TG2 only, being too large to enter the cell; consequently, it does not affect the intracellular roles of TG2, which is noteworthy given the important effects of intracellular TG2 on cell death, growth, and differentiation.^27^ The nomenclature IgG4P indicates that the hinge region of the IgG4 heavy chain sequence has a serine‐to‐proline substitution at position 241, which minimizes the possibility of Fab (antigen binding fragment)‐arm exchange.^28^ The structure of zampilimab has been previously described.^29^

In preclinical studies, using a model system where transforming growth factor β‐1 (TGFβ‐1) is used to drive accumulation of ECM proteins, zampilimab was effective in reducing TG2 activity, subsequent ECM accumulation in vitro and in vivo, and the development of fibrosis in a cynomolgus monkey unilateral ureteral obstruction (UUO) model.^29^ Pharmacokinetics (PK) of zampilimab were also explored in this study, and were as expected for the IgG4P format: zampilimab plasma concentration increased in a dose‐dependent manner, peaking at 6 h post‐dose in this preclinical model.^29^

Currently, four anti‐fibrotic therapies have received FDA approval: pirfenidone, nintedanib, nerandomilast, and tocilizumab.^30^, ^31^, ^32^, ^33^, ^34^ Pirfenidone, nintedanib, and nerandomilast are approved for the treatment of idiopathic pulmonary fibrosis (IPF).^30^, ^31^, ^33^, ^34^ Nintedanib is also approved for the treatment of other chronic fibrosing interstitial lung diseases (ILDs) with a progressive phenotype, as well as systemic sclerosis‐associated ILD (SSc‐ILD).^31^ In addition to IPF, nerandomilast is approved for progressive pulmonary fibrosis.^33^ Tocilizumab is approved for SSc‐ILD.^32^ Pirfenidone, nintedanib, nerandomilast, and tocilizumab act via different mechanisms from zampilimab: pirfenidone inhibits collagen synthesis and reduces fibroblast proliferation; nintedanib is a tyrosine kinase inhibitor targeting growth factor pathways; nerandomilast is a preferential phosphodiesterase 4B inhibitor that modulates inflammatory and fibrotic signaling; and tocilizumab is an anti‐interleukin (IL)‐6 receptor antibody.^35^, ^36^, ^37^, ^38^ None of these therapies has been shown to stop disease progression in pulmonary fibrosis, nor are they approved for the treatment of kidney fibrosis. Overall, there is a substantial unmet need for novel therapies for fibrotic diseases involving various organs.^39^, ^40^, ^41^, ^42^

In view of the role of TG2 in wound healing, a competitive immunofluorescence assay was developed, allowing accurate quantification of the target occupancy (TO) of zampilimab in the wounded skin of healthy participants.^43^ In a model of fibrosis (UUO in cynomolgus monkeys), this assay format demonstrated that the TO of zampilimab in the kidney was correlated with target engagement (TE; using an in situ TG activity assay) in both kidney and skin.^43^ Primate kidney TO/TE and skin TO also increased in a dose‐dependent manner with circulating levels of zampilimab.^43^ In a human skin biopsy study, the correlation of these results with ex vivo incubation of zampilimab, provided proof of principle for a ‘biopsy‐on‐biopsy’ skin model to indirectly estimate the TO of novel therapeutics.^43^

A Phase 1 first‐in‐human study (UP0029; NCT02879877) was conducted to investigate the safety, tolerability, and PK of intravenous (IV; up to 2000 mg) and subcutaneous (SC; up to 1000 mg) doses of zampilimab in healthy participants. One participant receiving IV zampilimab 2000 mg experienced a serious treatment‐emergent adverse event (TEAE) of moderate infusion‐related reaction, which met one of the stopping criteria defined in the protocol and resulted in a temporary study hold by the Safety Monitoring Committee (SMC). Although this event resolved within 3 days and the participant completed the follow up, no further dose escalation took place and the study was terminated.

In the early clinical development of novel drug candidates, focus is placed on safety while the optimal dosing regimen is not yet known, meaning development strategies (including dose refinement) sometimes need to be adapted based upon emergent information.^44^ With this in mind, a second Phase 1 study (UP0105; NCT04705350) was conducted to further investigate the safety, tolerability, and PK of two high‐dose levels of IV zampilimab (2000 and 3000 mg), with a lower rate of infusion than that used in the UP0029 study. Here, we describe the results from both Phase 1 studies, the rationale for the temporary study hold, and an overview of lessons learned from UP0029, which were applied to the UP0105 study.

## Methods

### UP0029—Phase 1 First‐in‐Human Study of Zampilimab at Single IV (10‐2000 mg) and SC (250‐1000 mg) Dose Levels

#### Study Design

A single‐center, randomized, participant‐ and investigator‐blind, placebo‑controlled study was conducted with healthy participants in a hospital setting (Northwick Park Hospital, Harrow, UK), in accordance with the International Council for Harmonisation (ICH) Good Clinical Practice (GCP) requirements, the ethical principles that have their origin in the principles of the Declaration of Helsinki, and local country laws. UP0029 is reported in accordance with the CONSORT reporting guidelines.^45^ Participants in the UP0029 study provided written informed consent to participate in the study. The study protocol, amendments, and participant‐informed consent forms for the UP0029 study were reviewed and approved by London—Bloomsbury Research Ethics Committee (approval number: 16/LO/0946).

The primary objective was to evaluate the safety and tolerability of zampilimab (single ascending IV doses up to 2000 mg and SC doses up to 1000 mg). Secondary objectives were to assess the PK (IV and SC) and the percent bioavailability of zampilimab (SC). Key exploratory objectives included the effect of zampilimab on TG2 expression and TO in skin wounds, and the immunogenicity of zampilimab, including the emergence of anti‐drug antibodies (ADAs).

Eligible participants were aged between 18 and 55 years, with a body mass index between 18.0 and 32.0 kg/m^2^ (body weight of 50‐90 kg). Participants were excluded if they had a known hypersensitivity to any components of zampilimab or placebo, or had received any prescription or non‐prescription medicines within 14 days or five half‐lives of the respective drug (whichever was longer). Occasional use of analgesics, such as paracetamol or ibuprofen, oral contraceptives, or inhaled corticosteroids for seasonal rhinitis was permitted. Key inclusion and exclusion criteria are shown in Table S1, Supporting Information.

Single doses of zampilimab were administered sequentially across ten cohorts (10‐2000 mg IV [Cohorts 1, 2, 4, 6, 8, and 9]; 250‐1000 mg SC [Cohorts 3, 5, 7, and 11]). Cohort 10 (IV 3000 mg) was not enrolled following the temporary study hold in Cohort 9 (2000 mg IV cohort) and subsequent protocol amendment. A further cohort, 500 mg SC (Cohort 11), which had been tested previously in Cohort 5 as a single dose with no safety concerns, was completed to further characterize the terminal elimination of the SC PK profile. Each cohort also included IV or SC administration of placebo, as appropriate. The UP0029 study design is shown in Figure S1, Supporting Information. All IV doses were administered as an infusion over 60 min at a maximum concentration of 100 mg/mL (maximum rate of infusion 33 mg/min). SC doses were administered as a fixed volume of 20 mL over 60 min, and the concentration was varied to achieve the desired dose. The study included a screening period of up to 3 weeks (Day −21 to Day −2), and a 6‐day in‐clinic period. This was followed by an ambulatory PK and safety follow‐up period of 67 or 115 days for Cohorts 1‐9 and Cohort 11, respectively. The maximum study duration was 14 weeks for all except the 500 mg SC cohorts (Cohorts 5 and 11; maximum duration 21 weeks).

While no formal sample size estimation was conducted, a sample size of up to 80 participants, who were randomized to ten cohorts (eight per cohort; six zampilimab and two placebo), was considered sufficient to meet the primary objective. An independent randomization statistician generated the randomization code and code break envelopes. Investigator site staff involved in the study were blinded to the assignments, with the exception of the pharmacist(s) who prepared and dispensed treatments and monitors who reviewed treatment‐related documentation. Zampilimab and placebo were not visually identical, so amber perfusor syringes and opaque tubing were used to blind administration. Within each cohort, a sentinel pair was incorporated (one zampilimab and one placebo); a blinded SMC review of the 72‐h post‐dose safety data of the given cohort was conducted ahead of dosing the remaining participants (who were randomized in a ratio of 5:1, zampilimab to placebo [Figure S1, Supporting Information]).

Following zampilimab administration, safety, tolerability, and PK parameters were assessed by an SMC, prior to each dose escalation. For the 10‐500 mg dose groups, escalation to the next dose level was permitted when ≥5 participants had been randomized (≥3 in the zampilimab group) and had completed 29 days of safety evaluations (and included all available PK data). Escalation to 1000/2000 mg was based on evaluation of up to 43 days of safety data and up to 29 days of PK data from the first five participants (three in the zampilimab group).

Safety‐related assessments included TEAEs, drug‐related TEAEs (as assessed by the investigator), discontinuations due to TEAEs, clinical laboratory tests, vital signs, electrocardiograms (ECGs), and physical examinations. TEAEs were defined as any event that was not present prior to the administration of zampilimab/placebo, or any unresolved event present before zampilimab/placebo administration that worsened in intensity following exposure to the treatment; TEAEs were coded using the Medical Dictionary for Regulatory Activities (MedDRA) v20.1. Drug‐related TEAEs were defined as those with a relationship of “related” or those with a missing relationship. Follow‐up for TEAEs continued for 71 days (Cohorts 1‐9) and 119 days (Cohort 11) after zampilimab/placebo administration, or for 30 days after withdrawal from the study. Safety variables were assessed at baseline and every visit until the final safety follow‐up visit (Day 72 [Cohorts 1‐9] or Day 120 [Cohort 11]), or at withdrawal from the study.

Blood samples for PK analysis were taken pre‐dose, immediately after the end of infusion/administration, and at various time points following the infusion/administration for each cohort up to 119 days (Day 120) post‐dose (Table S2, Supporting Information). PK parameters for zampilimab were calculated by non‐compartmental analysis methods and included maximum serum concentration (C_max_), time to maximum drug concentration (t_max_), area under the concentration–time curve from time 0 to infinity (AUC), AUC from 0 to last quantifiable concentration (AUC_(0‐t)_), and terminal half‐life (t_½_) after both IV and SC administration. Additional PK parameters were clearance (CL) and steady state volume of distribution (V_ss_) after IV administration, and apparent CL (CL/bioavailability [CL/F]) and apparent V_ss_ (V_ss_/F) after SC administration. PK parameters were calculated from individual serum concentration versus actual time profiles for zampilimab, evaluated after single doses. Volume of distribution (V_d_) was analyzed by the steady‐state method rather than the area method, as V_ss_ provides a more physiologically meaningful and comprehensive representation of drug distribution. In contrast, V_d(area)_ is derived from extrapolation of the log‐linear decline of the concentration–time profile and assumes instantaneous distribution—an assumption that may not be appropriate for drugs with slower tissue uptake.

Bioavailability was estimated as the ratio of the geometric means for AUC, according to the formula: F_absolute_ = AUC_sc_/AUC_IV_. The analyses were conducted separately for each dose level, at which the doses of SC and IV zampilimab were the same (250, 500, and 1000 mg), when the AUC was calculable. The analysis was implemented using the PROC MIXED procedure in SAS Version 9.3 or higher (SAS Institute, Cary, North Carolina, USA) on the log‐transformed data to obtain the treatment differences and the 95% confidence intervals (CI) of the differences.

Skin punch biopsies were obtained and used to assess TG2 expression and zampilimab TO, as demonstrated in the “biopsy‐on‐biopsy” skin model previously described.^43^ The timing of biopsy collection was informed by preclinical studies demonstrating that TG2 expression following skin injury is induced within 24 h and remains elevated for several days, with peak vascular TG2 immunoreactivity observed between Days 1 and 4 post‐wounding.^43^, ^46^ Accordingly, biopsies were collected at baseline, Day 2, and Day 4 (±24 h) for the 1000 mg SC and IV and 2000 mg IV cohorts; at baseline, Day 4 (±24 h), and Day 43 for the additional 500 mg SC cohort. TO was assessed using the competitive immunofluorescence assay (described in the bioanalytical methods).

The immunogenicity of single IV and SC doses of zampilimab was evaluated by assessing ADA levels on two occasions: at baseline and at the final safety follow‐up, or upon withdrawal from the study. ADAs were assessed using a three‐tiered assay approach: screening, confirmatory, and titration assays. Samples that were confirmed as positive in the confirmatory assay were further evaluated for titer. The sample collected on each occasion was reported as ADA positive if it tested positive in both the screening and confirmatory assays. A participant was considered overall treatment‐emergent ADA positive if they tested negative at baseline and positive at the post‐dose visit, or positive at baseline and at the post‐dose visit, with a four‐fold titer increase. A participant was considered overall treatment‐emergent ADA negative if they were ADA negative at both baseline and at post‐dose visit, or ADA positive at baseline and ADA negative at the post‐dose visit, or ADA positive at baseline and ADA positive at the post‐dose visit, with ≤4‐fold titer increase.

### Bioanalytical Methods

#### PK Assay

In human serum samples, zampilimab was measured using a validated bridging electrochemiluminescence immunoassay (ECLIA) assay on the Meso Scale Discovery (MSD) platform (Rockville, Maryland, USA), with a lower limit of quantification of 10.0 ng/mL. Biotinylated anti‐idiotype antibody (against zampilimab; produced and biotinylated by UCB) was immobilized on gold 96‐well MSD streptavidin plates, and zampilimab was detected with RuSULFO‐TAG anti‐idiotype antibody (against zampilimab; produced and tagged by UCB). The read buffer solution was then added to the wells and the plate was read using an MSD Sector Imager 6000. For each well, the intensity of the emitted luminescence was proportional to the amount of zampilimab present in the sample. Unknown concentrations were extrapolated from the standard curve fit with a 4PL regression using a 1/Y^2^ weighting factor. The intra‐ and inter‐run precision of the assay, expressed as percent relative standard deviation (RSD), were ≤3.2% and ≤5.6%, respectively. The intra‐ and inter‐run accuracy, calculated as the percent relative error (percentage of bias) of mean calibration measurements, were ≤│10.2│% and ≤│5.9│%, respectively.

#### ADA Assay

In human serum samples, ADAs were measured using an ECLIA assay on the MSD platform. The assay, which was developed and validated by UCB in 2017, consisted of a homogeneous bridging immunoassay using an acid dissociation step. The samples were first diluted in acetic acid at 1:10. One volume of those diluted samples was further diluted with four volumes of MasterMix (MM) containing biotinylated zampilimab and RuSULFO‐TAG zampilimab. The mix was incubated at room temperature for 2 h to allow the formation of immune complexes, which were then captured on an MSD streptavidin‐coated plate (previously blocked by UCB). After a final wash of the plate, read buffer was added, and the plate was read on an MSD Quickplex SQ120 reader. An electric current was applied to the bottom of the plate, leading to the emission of chemiluminescence, which was detected with a camera. The intensity of the chemiluminescence was proportional to the amount of anti‐zampilimab antibodies in the sample. The intra‐run precision, expressed as percent coefficient of variation (CV), was 1.87%, 3.62%, and 2.34% for the negative control, low positive control, and high positive control samples, respectively. The inter‐run precision was 0.65%, 7.35%, and 6.83% for the negative control, low positive control, and high positive control samples, respectively.

ADA detection was conducted following a tiered approach (screening–confirmation–titration). The assay was qualified, but drug tolerance characteristics could not be clinically evaluated due to the limited number of ADA samples collected. Additionally, during the cut point (CP) assessment, matrices with high background signals were excluded to obtain sufficiently low CP, which may have resulted in increased false positivity rates (FPRs) when using the study samples.

#### Competitive Immunofluorescence Assay

A competitive immunofluorescence assay was performed as previously described,^43^ to simultaneously identify both TG2 and zampilimab for the assessment of TO. Briefly, the assay used Alexa Fluor^®^ 546‐labeled zampilimab (red; UCB, UCB7858), and anti‐TG2 antibodies revealed with Alexa Fluor^®^ 488 secondary antibody (green; Life Technologies, A11017). The anti‐TG2 antibodies had binding epitopes remote from the zampilimab binding site (monoclonal antibodies [mAbs] IA12 and DH2). Co‐localization of Alexa Fluor^®^ 546‐labeled zampilimab and Alexa Fluor^®^ 488‐labeled anti‐TG2 antibodies resulted in a yellow fluorescence signal and demonstrated no TO. Where there is TO by unlabeled zampilimab administered in vivo, Alexa Fluor^®^ 546‐labeled zampilimab cannot bind ex vivo; only Alexa Fluor^®^ 488‐labeled anti‐TG2 antibodies can bind, and the resulting fluorescence signal is green.

Image analysis of skin sections was completed using a dedicated application developed by OracleBio on Visiopharm Software (Hoersholm, Denmark), which evaluated binding based on co‐localized fluorescent expression of red pixels (AlexaFluor^®^ 546‐labeled zampilimab) and green pixels (AlexaFluor^®^ 488‐labeled TG2 antibody) relative to total target expression and calculated the percentage TO.

### Statistical Methods

Statistical analyses were conducted using SAS^®^ Version 9.3 or later; all analysis on reported results were performed by treatment group. Categorical variables were summarized using frequency counts and percentages. Continuous data were summarized using numbers of participants, medians, and ranges. TO data were summarized graphically. The descriptive statistics for the PK data included the geometric CV and the geometric means.

The full analysis set (FAS)/safety analysis set (SAS) consisted of all randomized participants who received at least a partial dose of zampilimab or placebo. The PK “per protocol” set consisted of a subset from the FAS/SAS with no major protocol deviations that would affect PK parameters and with at least one evaluable zampilimab serum concentration/PK parameter (i.e., excluding participants receiving placebo).

## Results

### UP0029—Phase 1 First‐in‐Human Study of Zampilimab at Single IV (10‐2000 mg) and SC (250‐1000 mg) Dose Levels

#### Baseline Demographics

Between July 11, 2016 and January 31, 2018, 206 healthy participants were screened; of the 80 participants who were eligible and randomized, 76 (95%) completed the study (Figure S2, Supporting Information). Median age (FAS) was 35.5 years (range: 21‐55 years) and the majority were male (67%; Table 1). Overall, 41 randomized participants (53%) had previous/ongoing medical conditions, 30 (39%) had received a prior medication, and 46 (59%) received concomitant medications, with the most common being mupirocin, paracetamol, lidocaine, and xylocaine‐epinephrine (mupirocin, lidocaine, and xylocaine‐epinephrine were used with the skin biopsy sample collection in this study; Table S3, Supporting Information).

**Table 1:** Baseline Demographics of Participants in UP0029 (FAS)

	Zampilimab IV Administration							Zampilimab SC Administration				
	PBO IV n = 12	Cohort 1 10 mg n = 6	Cohort 2 50 mg n = 6	Cohort 4 250 mg n = 6	Cohort 6 500 mg n = 6	Cohort 8 1000 mg n = 6	Cohort 9 2000 mg n = 5	PBO SC n = 8	Cohort 3 250 mg n = 6	Cohorts 5/11^a^ 500 mg n = 11	Cohort 7 1000 mg n = 6	All participants N = 78
Median age, years (range)	36.5 (23‐53)	34.0 (21‐52)	37.0 (29‐43)	34.0 (22‐54)	40.0 (33‐45)	33.5 (22‐40)	42.0 (29‐51)	35.5 (27‐53)	24.5 (22‐46)	40.0 (22‐55)	39.0 (25‐52)	35.5 (21‐55)
Male, n (%)	7 (58)	6 (100)	4 (67)	5 (83)	5 (83)	4 (67)	1 (20)	6 (75)	5 (83)	8 (73)	1 (17)	52 (67)
Median BMI, kg/m^2^ (range)	24.2 (20‐27)	24.4 (19‐26)	24.7 (22‐28)	24.3 (22‐26)	23.9 (21‐28)	25.3 (22‐28)	24.1 (22‐28)	23.9 (20‐27)	24.6 (21‐29)	23.0 (20‐28)	24.4 (18‐31)	24.2 (18‐31)
Race, n (%)												
Asian	1 (8)	1 (17)	0	1 (17)	2 (33)	1 (17)	2 (40)	1 (13)	1 (17)	0	0	10 (13)
Black or African American	2 (17)	0	1 (17)	0	1 (17)	1 (17)	1 (20)	0	0	2 (18)	2 (33)	10 (13)
White	7 (58)	5 (83)	3 (50)	5 (83)	3 (50)	4 (67)	2 (40)	3 (38)	5 (83)	7 (64)	4 (67)	48 (62)
Other/mixed	2 (17)	0	2 (33)	0	0	0	0	4 (50)	0	2 (18)	0	10 (13)

BMI, body mass index; FAS, full analysis set; IV, intravenous; PBO, placebo; SC, subcutaneous; SMC, Safety Monitoring Committee.

a Cohort 11 was added following the study hold, as the SMC recommended a repeat SC dose of 500 mg.

#### Safety

Overall, 47 participants received a single IV infusion of zampilimab (n = 35) or placebo (n  =  12), and 31 received a single SC administration of zampilimab (n = 23) or placebo (n  =  8). At least one TEAE occurred in 72.4% of the participants receiving zampilimab (n  =  42/58) and 70% of those receiving placebo (n = 14/20; Table 2); all were mild to moderate in intensity. The most frequent TEAEs with zampilimab were nasopharyngitis (n  =  12; 20.7%), headache (n = 6; 10.3%), influenza‐like illness, and procedural pain (n = 4; 6.9%, each). For placebo, the most frequent TEAEs were nasopharyngitis (n = 6; 30%) and headache (n = 4; 20%). TEAEs reported by ≥2 participants are presented in Table S4, Supporting Information.

**Table 2:** Overview of TEAEs Reported for ≥1 Participants in Any Treatment Group in UP0029 (FAS)

	Zampilimab IV Administration							Zampilimab SC Administration					
n (%), [#]	PBO IV n = 12	Cohort 1 10 mg n = 6	Cohort 2 50 mg n = 6	Cohort 4 250 mg n = 6	Cohort 6 500 mg n = 6	Cohort 8 1000 mg n = 6	Cohort 9 2000 mg n = 5	PBO SC n = 8	Cohort 3 250 mg n = 6	Cohorts 5/11^a^ 500 mg n = 11	Cohort 7 1000 mg n = 6	PBO total n = 20	Zampilimab total n = 58
Any TEAEs	9 (75) [23]	3 (50) [3]	4 (66.7) [9]	4 (66.7) [9]	5 (83.3) [6]	5 (83.3) [19]	4 (80) [8]	5 (62.5) [8]	4 (66.7) [7]	7 (63.6) [20]	6 (100) [13]	14 (70) [31]	42 (72.4) [94]
Serious TEAEs	0	0	0	0	0	0	1 (20) [1]	0	0	0	0	0	1 (1.7) [1]
Study discontinuations due to TEAEs	0	0	0	0	0	0	0	0	0	0	0	0	0
Drug‐related TEAEs	3 (25) [6]	0	1 (16.7) [1]	2 (33.3) [2]	1 (16.7) [1]	3 (50) [4]	2 (40) [2]	0	1 (16.7) [1]	2 (18.2) [4]	4 (66.7) [8]	3 (15) [6]	16 (27.6) [23]
Severe TEAEs	0	0	0	0	0	0	0	0	0	0	0	0	0
Deaths^b^	0	0	0	0	0	0	0	0	0	0	0	0	0

TEAEs were defined as any event that was not present prior to the administration of zampilimab/PBO, or any unresolved event present before administration that worsened in intensity following exposure to the treatment; TEAEs were coded using MedDRA v20.1. Drug‐related TEAEs were defined as those with a relationship of ‘related’ or those with missing relationship.

FAS, full analysis set; IV, intravenous; MedDRA, Medical Dictionary for Regulatory Activities; PBO, placebo; PK, pharmacokinetics; SC, subcutaneous; SMC, Safety Monitoring Committee; TEAE, treatment‐emergent adverse event.

a Cohort 11 was added following the study hold, as the SMC recommended a repeat SC dose of 500 mg to further characterize the SC PK profile.

b Deaths: all deaths and TEAEs leading to death.

[#] is the individual occurrences of the defined TEAE.

Drug‐related TEAEs occurred in 16 (27.6%) and three (15%) participants with zampilimab and placebo, respectively (Table 2). The most frequent drug‐related TEAEs were headache (n = 4; 6.9%), infusion‐related reaction, nasal congestion, and hot flush (n  =  2; 3.4%, each) with zampilimab, and headache (n = 2; 10%) with placebo. Drug‐related TEAEs reported by ≥2 participants are presented in Table S4, Supporting Information. Most drug‐related TEAEs occurred in the zampilimab 1000 mg IV group (n = 3; 50%) and zampilimab 1000 mg SC group (n = 4; 66.7%); all were mild to moderate.

There were no clinically relevant abnormalities on ECGs, vital signs, or laboratory parameters following zampilimab administration. One TEAE associated with abnormal vital signs (pyrexia) occurred with zampilimab 1000 mg IV.

Two infusion‐related reactions were reported, the first being a moderate TEAE with zampilimab 500 mg IV and the second a moderate TEAE with zampilimab 2000 mg IV. The first event (in the 500 mg IV group) occurred 38 h after single‐dose administration and was accompanied by an increase in C‐reactive protein; the participant was treated with paracetamol and the event resolved in 3 days with no sequelae. The second event (in the zampilimab 2000 mg IV group) was a serious TEAE, which met a predefined protocol criterion for a study hold. Symptoms, which included pruritus, itchy eyes, chest tightness, and cough, occurred 5 min after initiation of the infusion and led to premature discontinuation of zampilimab after 720 mg (36% of the 2000 mg dose) had been administered (rate of infusion 33 mg/min). The participant was observed closely, and no significant changes in vital signs were recorded. The initial symptoms of itchy eyes, chest tightness, and coughing resolved within the same day, with no treatment given on the first day; all other symptoms resolved within 3 days. Additional symptoms of headache, tiredness, and low back pain were reported within 3‐8 h from the start of infusion. On the second day, following zampilimab administration, the participant was treated with 1 g paracetamol for the headache, which was associated with the infusion reaction, and went on to complete the safety follow‐up period. In addition, in the 2000 mg IV cohort, another study participant developed a mild lip swelling after completion of the IV infusion, which resolved spontaneously with no further symptoms. Although not reported as such, it is possible that this event represented a mild infusion reaction. The high concentration of zampilimab (100 mg/mL) and the high rate of infusion (33 mg/min) over 60 min were considered to be contributing factors to the infusion reaction. No further dosing in the 2000 mg IV cohort and no further dose escalation occurred in this study.

Of the 80 randomized participants, four (5%) discontinued (Figure S2, Supporting Information); the reasons were: withdrawal of consent (n = 1); pre‐existing tinea versicolor (n = 1); other reason (personal reasons; n  =  1). When the study was temporarily suspended, one participant in the 2000 mg IV cohort was randomized but did not receive any treatment (n = 1). No deaths occurred during the study.

#### Pharmacokinetics

Following IV and SC administration, serum zampilimab exposure (C_max_ and AUC) was approximately dose‐proportional across the dose range (500‐2000 mg). Maximum zampilimab concentrations were generally reached at the end of the infusion. Across the zampilimab SC cohorts, t_max_ was reached after 4‐7 days on average (range: 4‐15 days) and appeared to be independent of dose. Where it was possible to derive, the t_1/2_ of zampilimab was estimated to be 17‐23 days across both routes of administration. On average, CL and V_ss_ were reported to be 0.209 L/day and 5.77 L, respectively, for the 1000 mg IV dose and 0.152 L/day and 3.76 L, respectively, for the 2000 mg IV dose. Following a 1000 mg dose, absolute bioavailability for the SC formulation was 77.2% (95% CI: 60.3, 98.9). A summary of all PK endpoints for this study is provided in Table 3.

**Table 3:** Serum PK Parameters of UP0029 (PK‐Per Protocol Set)

		Zampilimab IV Administration						Zampilimab SC Administration			
Parameter	Statistic	Cohort 1 10 mg n = 6	Cohort 2 50 mg n = 6	Cohort 4 250 mg n = 6	Cohort 6 500 mg n = 6	Cohort 8 1000 mg n = 6	Cohort 9 2000 mg n = 5	Cohort 3 250 mg n = 6	Cohort 5 500 mg n = 6	Cohort 11^a^ 500 mg n = 5	Cohort 7 1000 mg n = 6
AUC_(0‐∞)_ (day·µg/mL)	Geo mean (Geo CV, %)	NA	NA	NA	NA	4782 (16.5)	13,200 (28.5)	NA	NA	1901 (7.6)	3693 (20.1)
	Mean (SD)	NA	NA	NA	NA	4838 (829)	13,610 (4050)	NA	NA	1905 (146)	3752 (745)
AUC_(0‐t)_ (day·µg/mL)	Geo mean (Geo CV, %)	21.3 (12.1)	102 (15.6)	506 (11.8)	1041 (15.4)	3685 (14.5)	10,660 (29.2)	234 (34.6)	558 (34.8)	1863 (7.5)	2088 (57.3)
	Mean (SD)	21.5 (2.63)	103 (15.9)	509 (57.4)	1051 (162)	3719 (565)	11,010 (3440)	245 (80.2)	584 (183)	1867 (144)	2296 (898)
C_max_ (µg/mL)	Geo mean (Geo CV, %)	3.44 (13)	15.2 (20.5)	77.4 (18)	156 (17.4)	307 (10.6)	866 (25.1)	21.2 (29.6)	47.9 (36.5)	56.7 (19)	93.4 (30.4)
	Mean (SD)	3.46 (0.449)	15.4 (2.86)	78.4 (13.8)	158 (25.4)	308 (32.2)	888 (240)	22 (6.52)	50.3 (16.1)	57.6 (11.6)	96.9 (29)
t_max_ (day)	Median (min, max)	0.042 (0.042, 0.124)	0.042 (0.042, 0.126)	0.124 (0.042, 0.126)	0.208 (0.042, 0.377)	0.042 (0.042, 0.438)	0.042 (0.042, 0.042)	7.03 (4.03, 14.3)	7.05 (4.03, 15.0)	4.03 (4.03, 7.26)	5.52 (3.03, 7.00)
t_1/2_ (day)	Geo mean (Geo CV, %)	NA	NA	NA	NA	20.6 (13.7)	17.9 (6.8)	NA	NA	20.2 (14.6)	22.6 (14.9)
	Mean (SD)	NA	NA	NA	NA	20.8 (2.96)	17.9 (1.26)	NA	NA	20.4 (2.85)	22.8 (3.36)
CL (L/day)	Geo mean (Geo CV, %)	NA	NA	NA	NA	0.209 (16.5)	0.152 (28.5)	NA	NA	NA	NA
	Mean (SD)	NA	NA	NA	NA	0.211 (0.0332)	0.156 (0.0404)	NA	NA	NA	NA
V_ss_ (L)	Geo mean (Geo CV, %)	NA	NA	NA	NA	5.77 (12.2)	3.76 (29.9)	NA	NA	NA	NA
	Mean (SD)	NA	NA	NA	NA	5.80 (0.699)	3.87 (0.983)	NA	NA	NA	NA
CL/F (L/day)	Geo mean (Geo CV, %)	NA	NA	NA	NA	NA	NA	NA	NA	0.263 (7.6)	0.271 (20.1)
	Mean (SD)	NA	NA	NA	NA	NA	NA	NA	NA	0.264 (0.0196)	0.275 (0.0546)
V_ss_,/F (L)	Geo mean (Geo CV, %)	NA	NA	NA	NA	NA	NA	NA	NA	7.68 (14.7)	8.84 (25.1)
	Mean (SD)	NA	NA	NA	NA	NA	NA	NA	NA	7.75 (1.14)	9.05 (2.16)

AUC_(0‐∞)_, area under the concentration‐time curve from time 0 to infinity; AUC_(0‐t)_, AUC from 0 to last quantifiable concentration; CL, clearance; C_max_, maximum serum concentration; CV, coefficient of variation; F, bioavailability; Geo, Geometric; IV, intravenous; max, maximum; min, minimum; NA, not applicable; PK, pharmacokinetics; SC, subcutaneous; SD, standard deviation; SMC, Safety Monitoring Committee; t_1/2_, terminal half‐life; t_max_, time to maximum drug concentration; V_ss_, steady state volume of distribution.

a Cohort 11 was added following the study hold, as the SMC recommended a repeat SC dose of 500 mg.

The study participant who experienced an infusion‐related reaction received 36% of their 2000 mg dose before the infusion was stopped. Serum zampilimab concentrations were 215 µg/mL and 230 µg/mL at the end of the infusion and at the 8‐h nominal time point, respectively. This was lower than the average C_max_ reported for the 2000 mg dose group (geometric mean of 866 µg/mL; the participant was excluded from the PK per‐protocol set analysis).

#### TG2 Occupancy and Expression

**Figure 1:**
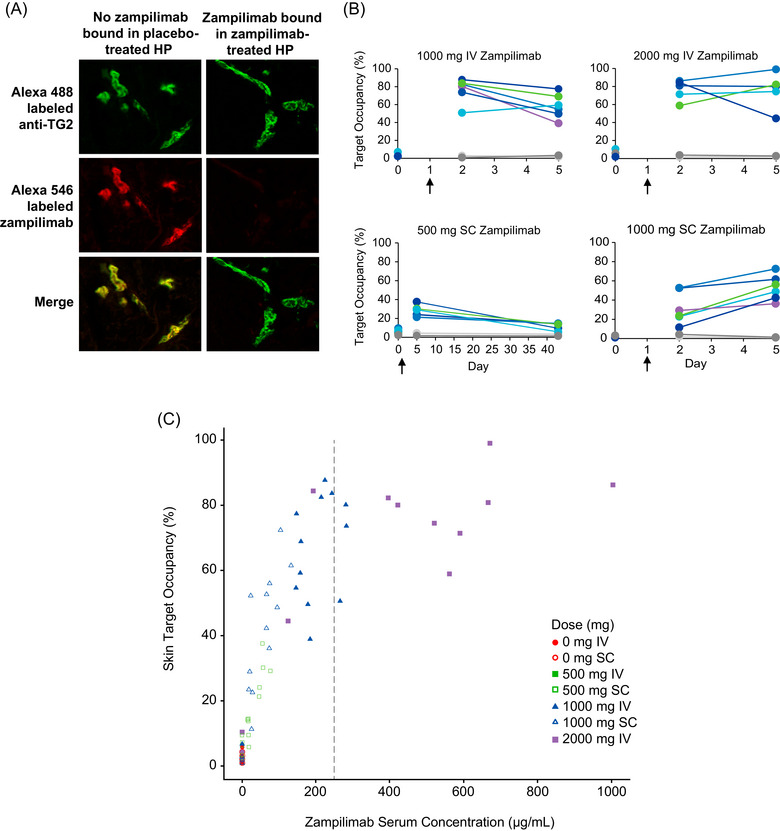
Zampilimab TO in human skin. (A) Exemplar images from participants receiving placebo (where both Alexa Fluor^®^ 488‐labeled “total” TG2 and Alexa Fluor^®^ 546‐labeled zampilimab bound equally—yellow merge [left‐hand image]) and zampilimab‐treated (where Alexa Fluor^®^ 546‐labeled zampilimab was prevented from binding by prior in vivo zampilimab binding—green merge [right‐hand image]) demonstrating high TO. (B) Following dosing (black arrows) of IV or SC zampilimab, TO based on Alexa Fluor^®^ 546/488 co‐localization was assessed per participant, each line represents mean data per individual, placebo‐treated participants are in gray. The navy blue line for 2000 mg IV zampilimab with lower TO represents the participant whose infusion was stopped due to the infusion‐related reaction. (C) A dose‐dependent relationship between skin TO and zampilimab serum concentration was observed with maximal occupancy achieved at concentrations >250 µg/mL. HP, healthy participant; IV, intravenous; SC, subcutaneous; TG2, Transglutaminase 2; TO, target occupancy.

Zampilimab TO was observed in human skin following dosing (Figure 1A); and TG2 staining was localized to blood vessels in the dermal wound biopsies and showed a dose‐response relationship with TO increasing with dose. TO appeared to be close to maximal at Day 2 with 1000 and 2000 mg IV doses and was up to 99% on Day 5. There was an increase in TO of up to 73% for all participants who received 1000 mg SC from Day 2 to Day 5 (Figure 1B). TO in skin was directly dependent on serum zampilimab concentration, with maximal occupancy achieved at concentrations of approximately 250 µg/mL or higher (Figure 1C).

#### Anti‐Drug Antibodies

In total, 20 participants (26%; zampilimab, n = 15; placebo, n = 5) were ADA positive at baseline. With zampilimab, 20 participants (36%; IV, n = 13; SC, n = 7) had developed a treatment‐emergent antibody response by Day 72/120. There was no impact of ADA on serum concentrations of zampilimab. The study participant whose infusion was stopped due to an infusion‐related reaction was, overall, treatment‐emergent ADA negative.

Overall, due to the low number of ADA samples collected, the observed incidence rate was inconclusive as the drug tolerance characteristics of the ADA bioanalytical method could not be clinically evaluated. Consequently, the use of this method may have affected the reported ADA positivity rate and the overall impact of immunogenicity on the PK. Therefore, the ADA method was further optimized for future studies, including UP0105.

#### Study Termination and Subsequent PK Modeling

The target zampilimab trough serum concentration required for maximum TG2 occupancy was determined using pharmacologically relevant data from nonclinical studies and data from healthy participants obtained in the UP0029 study. Nonclinical data suggested that high levels of TG2 enzyme inhibition were likely to be required to inhibit function. As we have reported, the relationship between percentage TO (normalized) and zampilimab serum concentration indicated that maximal occupancy was achieved at serum concentrations of approximately 250 µg/mL or higher. The temporary study hold occurred after 47 participants had been dosed, and following completion of Cohort 11 (500 mg SC), the study was terminated. Subsequent PK modeling of the UP0029 study IV cohorts indicated that zampilimab doses of 2000 or 3000 mg were required to achieve a target trough serum concentration of 250 µg/mL at steady state and at least 75% TG2 occupancy in >50% of participants (maximum TO achievable was around 80%). Following the results obtained from the UP0029 study and the subsequent PK modeling, high single IV doses of zampilimab were explored further, using an adapted dosing administration strategy to decrease the likelihood of future infusion‐related reactions.

## Methods

### UP0105—Phase 1 Safety and Tolerability Study of Zampilimab at Single 2000 and 3000 mg IV Doses in Healthy Participants

#### Study Design

UP0105 was a randomized, participant‐ and investigator‐blind placebo–controlled study, conducted in healthy participants in a medicines research center (Hammersmith Medicines Research, London, UK). Ethical considerations for UP0105 and consent to participate were as described for the UP0029 study. The study protocol, amendments, and participant‐informed consent forms for the UP0105 study were reviewed and approved by London—Hampstead Research Ethics Committee (approval number: 20/LO/1192).

The primary objective was to evaluate the safety and tolerability of a single IV infusion of zampilimab (2000 and 3000 mg). The secondary objective was to characterize the PK of IV zampilimab (single doses). The exploratory objectives were to evaluate the effect of single IV doses of zampilimab on blood TG2 levels and to assess the immunogenicity of zampilimab. Key eligibility criteria for this study were the same as the UP0029 study (Table S1, Supporting Information).

While no formal sample size estimation was conducted, at least 32 individuals were screened in order to enroll a maximum of 16 participants (eight per cohort; six zampilimab and two placebo), randomized in a 3:1 ratio (zampilimab:placebo) to receive a single IV dose. Participants were assigned a unique randomization number in ascending numerical order by the study center, which determined the participant's assignment to receive zampilimab or placebo. A sentinel pair (one zampilimab and one placebo) was incorporated into the treatment assignment of each cohort, with participants being closely monitored for infusion reactions. Blinded infusion bags were labeled with each study participant's unique randomization number and dispensed on Day 1 of the treatment period. Investigators and participants remained blinded to the assigned group throughout the study. The possibility of some sponsor staff becoming unblinded to the assignment cannot be excluded; however, this was minimized and would not have included sponsor staff who were in contact with the study center, unless required to maintain participant safety.

The study included a screening period of up to 3 weeks (Day −21 to Day −2) and a 5‐day in‐clinic period, followed by a 67‐day safety follow‐up and an end‐of‐study visit on Day 120. Single IV doses of zampilimab (2000 or 3000 mg) were administered across two sequential cohorts (in each cohort, two participants received IV placebo [Figure S3, Supporting Information]). Using the lessons learned from the UP0029 study administration strategy, the concentration of zampilimab was reduced to a maximum of 10 mg/mL and the infusion period was increased from 60 to 120 min (maximum rate of infusion 25 mg/min).

An SMC reviewed blinded safety data for the sentinel pairs, obtained for up to 72 h after administration of zampilimab or placebo, and a safety assessment was made before subsequent participants in the cohort received IV zampilimab. A blinded review of safety data from Day 1‐29 and PK data from Day 1‐15 (for at least six participants in the zampilimab 2000 mg group), was conducted before the dose was escalated to 3000 mg IV (single doses).

The primary safety variables were the incidence and intensity of TEAEs. Other safety assessments included: clinical laboratory tests, vital signs, ECGs, physical examination, and local tolerability (assessment of the IV infusion site). TEAEs were defined as previously described for the UP0029 study. Follow‐up for TEAEs continued for 119 days (Day 120) after zampilimab administration. The other safety variables were assessed at baseline, during the treatment period (Days 1‐5), during the safety follow‐up (Days 8, 15, 22, 29, 43, and 72), and on Day 120, or upon withdrawal from the study.

Blood samples for PK analysis were taken pre‐dose, immediately after the end of the infusion/administration, and at various time points following the infusion/administration for each cohort up to 119 days post‐dose (Day 120; Table S5, Supporting Information). PK parameters were calculated by non‐compartmental analysis methods and included C_max_, t_max_, AUC, AUC_(0‐t)_, t_½_, and CL, which were evaluated after a single IV dose of zampilimab.

ADAs to zampilimab were evaluated in blood samples collected from all participants on the pre‐dose visit and Days −1 (baseline), 15, 29, 43, 72, and 120. ADAs were assessed as in the UP0029 study.

### Bioanalytical Methods

#### PK Assay

Zampilimab was measured in serum samples using the same validated bridging ECLIA assay as described for the UP0029 study, except that the anti‐idiotypic antibody used for capture and detection differed from that of UP0029. Biotinylated anti‐idiotype antibody (against zampilimab; produced and biotinylated by UCB) at 0.125 µg/mL was immobilized on gold 96‐well MSD streptavidin Quickplex plates and zampilimab was detected with RuSULFO‐TAG anti‐idiotype antibody (against zampilimab; produced and tagged by UCB) at 0.25 µg/mL. The read buffer solution was then added to the wells, and the plate was read using an MSD Quickplex SQ120 reader. The intra‐ and inter‐run precision of the assay, as expressed by the percentage RSD, were ≤4.0% and ≤4.6%, respectively. The inter‐run accuracy, calculated as the bias of mean quality control measurements, was ≤│6.5│%.

#### TG2 Assay

In human ethylenediaminetetraacetic acid (EDTA) plasma samples from the UP0105 study, a TG2 signature peptide (representative of total TG2) was measured using a validated immunoprecipitation liquid chromatography‐mass spectrometry/mass spectrometry (IP‐LC‐MS/MS) assay, using a Waters Acquity UPLC Peptide BEH C18 Colum (300 Ä, 2.1×100 mm, 1.7 µm), with a lower limit of quantification of 1.00 ng/mL. Mobile Phase A and B were comprised of H_2_O:ACN:acetic acid; 95:5:0.1 and H_2_O:ACN:acetic acid; 5:95:0.1, respectively. Samples were mixed with biotinylated anti‐TG2 capture agent (produced and biotinylated by UCB) and magnetic beads and washed on a Kingfisher automated device; beads were mixed with an internal standard TG2‐PS‐SI +8 isotopically labeled peptide (Eurogentec, Liège, Belgium). Samples were then denatured using Tris(2‐carboxyethyl)phosphine. Following this, iodoacetamide was added to prevent re‐association of the reduced cysteine residues and L‐cysteine was added to remove the excess iodoacetamide. The denatured samples were then enzymatically digested with trypsin and evaluated using LC‐MS/MS analysis. The endogenous TG2 (TG2 signature peptide) and internal standard were analyzed by reverse phase LC and detected by electrospray mass spectrometry (ESI/MS/MS) using multiple reaction monitoring in positive ionization mode. Transitions for TG2 signature peptide and the internal standard were *m*/*z* 365.3 → 529.4 and 369.3 → 537.4, respectively. Intra‐ and inter‐run precision, expressed as percentage RSD, were ≤4.5% and ≤2.4%, respectively. Inter‐run accuracy, expressed as percent relative error of mean quality control measurements, was ≤|4.6|%.

#### ADA Assay

For the UP0105 study, an ADA assay was developed with an improved statistical CP determination strategy (using an iterative mixed‐effects model and statistical analysis considering the distribution of the data). This assay, validated by UCB in 2021, resulted in optimal FPRs and demonstrated good sensitivity balanced with appropriate drug tolerance.

The assay consisted of a homogeneous bridging immunoassay on the MSD Quickplex SQ120 platform. The samples were diluted at 1 in 5 with the assay diluent followed by another dilution with 175 mM glycin (pH 1.5). After a 1‐h acidification time, one volume of acidified samples was neutralized with three volumes of MM solution, containing biotinylated zampilimab and RuSULFO‐TAG zampilimab diluted in a mix of 1.5 M Tris pH 9.5 and assay diluent (screening assay), or biotinylated zampilimab, RuSULFO‐TAG zampilimab, and unconjugated zampilimab diluted in a mix of 1.5 M Tris pH 9.5 and assay diluent (confirmatory assay). Capture and detection reagents were added at a 1:1 molar ratio to reach a final concentration of 2 µg/mL after minimal required dilution (MRD). Unconjugated zampilimab was added at a final concentration of 100 µg/mL after MRD. The final MRD was 1/100. This mix was incubated at room temperature for 2 h to allow the formation of immune complexes, which were captured on gold 96‐well MSD streptavidin Quickplex plates (previously blocked by UCB). The read buffer solution was added to the wells and the plate was read with the MSD Quickplex SQ120 reader. For each well, the intensity of the emitted luminescence was proportional to the amount of anti‐zampilimab antibodies present in the sample. The screening assay intra‐run precision, expressed as percent CV, was 6.52%, 6.90%, 5.02%, and 8.08% for the negative control, low positive control, medium positive control, and high positive control samples, respectively. The screening assay inter‐run precision was 11.02%, 10.29%, 11.70%, and 11.31% for the negative control, low positive control, medium positive control, and high positive control samples, respectively. The confirmatory assay intra‐run precision was 8.96%, 6.22%, 0.131%, and 0.0410% for the negative control, low positive control, medium positive control, and high positive control samples, respectively. The confirmatory assay inter‐run precision was 9.31%, 6.60%, 0.17%, and 0.0452% for the negative control, low positive control, medium positive control, and high positive control samples, respectively.

A direct comparison between the ADA results of the UP0029 and UP0105 studies was not feasible due to differences in the validation methods and assay characteristics used in each study.

#### Competitive Immunofluorescence Assay

The same competitive immunofluorescence assay as described for the UP0029 study was performed to identify TG2 and zampilimab simultaneously for the assessment of TO. Image analysis of skin sections was completed using the same application as described for the UP0029 study.

### Statistical Methods

The same statistical methods outlined for the UP0029 study were used. The participants that made up the FAS, SAS, and PK ‘per protocol’ set are as described for UP0029.

## Results

### UP0105—Phase 1 Safety and Tolerability Study of Zampilimab at Single 2000 and 3000 mg IV Doses in Healthy Participants

#### Baseline Demographics

Between January 11, 2021 and July 28, 2021, 16 participants were randomized to receive zampilimab 2000 mg (n = 6), zampilimab 3000 mg (n = 6), or placebo (n = 4). Median age (SAS) was 30.5 years (range: 19‐53 years) and most were male (81.3%; Table 4). There were 22 screening failures (Figure S4, Supporting Information). A summary of medical history and current medications is provided in Table S6, Supporting Information.

**Table 4:** Baseline Demographics of Participants in UP0105 (SAS)

	PBO IV Total n = 4	Zampilimab IV 2000 mg n = 6	Zampilimab IV 3000 mg n = 6	Zampilimab IV Total n = 12	All participants N = 16
Median age, years (range)	27.0 (27‐30)	39.0 (25‐53)	33.0 (19‐39)	33.0 (19‐53)	30.5 (19‐53)
Male, n (%)	3 (75)	5 (83.3)	5 (83.3)	10 (83.3)	13 (81.3)
Median BMI, kg/m^2^ (range)	22.9 (21.4‐28.9)	22.8 (19.8‐24.5)	25.6 (20.3‐28.7)	24.4 (19.8‐28.7)	24.2 (19.8‐28.9)
Race, n (%)					
Asian	1 (25)	1 (16.7)	0	1 (8.3)	2 (12.5)
Black or African American	1 (25)	0	0	0	1 (6.3)
White	1 (25)	4 (66.7)	5 (83.3)	9 (75)	10 (62.5)
Other/mixed	1 (25)	1 (16.7)	1 (16.7)	2 (16.7)	3 (18.8)

BMI, body mass index; IV, intravenous; PBO, placebo; SAS, safety analysis set.

#### Safety

All 16 randomized participants received a single dose of zampilimab or placebo. At least one TEAE was reported by eight participants (66.7%) with zampilimab and one (25%) with placebo (Table 5); all were mild to moderate in intensity and none were drug‐related. The most frequent TEAEs in the zampilimab total group were headache (n = 3; 25%), increased blood creatine phosphokinase (CPK), increased blood lactate dehydrogenase (LDH), and increased aspartate aminotransferase (AST; all n = 2; 16.7%); all resolved spontaneously. With placebo, the only TEAE was a bruise at the catheter site (n = 1; 25%). All TEAEs are presented in Table S7, Supporting Information.

**Table 5:** Overview of TEAEs Reported for ≥1 Participants in Any Treatment Group in UP0105 (SAS)

n (%), [#]	PBO IV Total n = 4	Zampilimab IV 2000 mg n = 6	Zampilimab IV 3000 mg n = 6	Zampilimab IV Total n = 12
Any TEAEs	1 (25) [1]	3 (50) [4]	5 (83.3) [14]	8 (66.7) [18]
Serious TEAEs	0	0	0	0
Study discontinuations due to TEAEs	0	0	0	0
Drug‐related TEAEs	0	0	0	0
Severe TEAEs	0	0	0	0
Deaths^a^	0	0	0	0

TEAEs were defined as any event that was not present prior to the administration of zampilimab/PBO, or any unresolved event present before administration that worsened in intensity following exposure to the treatment; TEAEs were coded using MedDRA v20.1. Drug‐related TEAEs were defined as those with a relationship of ‘related’ or those with missing relationship.

IV, intravenous; MedDRA, Medical Dictionary for Regulatory Activities; PBO, placebo; SAS, safety analysis set; TEAE, treatment‐emergent adverse event.

a Deaths: all deaths and TEAEs leading to death.

[#] is the individual occurrences of the defined TEAE.

No clinically significant abnormalities related to zampilimab were observed in hematology, clinical chemistry, or urinalysis laboratory results. Similarly, no clinically significant changes were observed in vital signs, ECG parameters, or physical examinations following administration of zampilimab.

The incidence of TEAEs was lower in the zampilimab 2000 mg group (n = 3; 50%) than in the 3000 mg group (n = 5; 83.3%). The only TEAE occurring in more than one participant receiving zampilimab 2000 mg was headache (n = 2; 33.3%). The TEAEs that occurred in more than one participant receiving zampilimab 3000 mg were increased AST, increased CPK, and increased LDH (all n = 2; 33.3%). For both participants receiving 3000 mg who had abnormal blood chemistry values (increased AST and increased CPK), the investigator noted that these changes had been caused by exercise and were not related to the administration of zampilimab. No serious TEAEs were reported in this study. There were no TEAEs leading to discontinuation and no deaths were reported in this study.

#### Pharmacokinetics and Pharmacodynamics

Following IV dosing of zampilimab, serum zampilimab exposure (C_max_ and AUC) increased between the 2000 and 3000 mg groups, but in a slightly less than dose‐proportional manner. t_max_ was reached 2.08 h (median) after the start of the infusion for both zampilimab 2000 and 3000 mg. Maximum zampilimab concentrations were generally reached at the end of the infusion, at a median of 2.08 h after the start of the infusion for both 2000 and 3000 mg. On average, the t_1/2_ of zampilimab was estimated to be 22.73 days (range: 19.7‐25.1 days) for 2000 mg and 19.58 days (range: 10.9‐24.0 days) for 3000 mg. CL was 0.19 L/day for the 2000 mg IV dose and 0.23 L/day for the 3000 mg IV dose. Following single IV administration, plasma concentrations of TG2 were consistent between doses of 2000 and 3000 mg. A summary of PK endpoints for this study is presented in Table 6.

**Table 6:** Serum PK Parameters of UP0105 (PK‐Per Protocol Set)

		Zampilimab IV Administration	
Geometric Mean	Statistic	2000 mg n = 5^a^	3000 mg n = 6
AUC_(0‐∞)_ (day·µg/mL)	Geo mean (Geo CV, %)	10,817 (24.1)	13,028 (14.4)
	Mean (SD)	11,056 (2527)	13,141 (1919)
AUC_(0‐t)_ (day·µg/mL)	Geo mean (Geo CV, %)	10,561 (23.5)	12,782 (13.8)
	Mean (SD)	10,783 (2409)	12,885 (1802)
C_max_ (µg/mL)	Geo mean (Geo CV, %)	718 (19.5)	910 (20.2)
	Mean (SD)	728 (136)	927 (204)
t_max_ (day)	Median (min, max)	2.08 (2.08, 2.12)	2.08 (2.08, 4.00)
t_1/2_ (day)	Geo mean (Geo CV, %)	22.70 (10.1)	19.58 (30.1)
	Mean (SD)	22.8 (2.27)	20.2 (4.79)
CL (L/day)	Geo mean (Geo CV, %)	0.18 (24.5)	0.23 (14.4)
	Mean (SD)	0.189 (0.0472)	0.232 (0.0325)

AUC_(0‐∞)_, area under the concentration–time curve from time 0 to infinity; AUC_(0‐t)_, AUC from 0 to last quantifiable concentration; CL, clearance; C_max_, maximum serum concentration; CV, coefficient of variation; Geo, geometric; IV, intravenous; max, maximum; min, minimum; PK, pharmacokinetics; SC, subcutaneous; SD, standard deviation; t_1/2_, terminal half‐life; t_max_, time to maximum drug concentration.

a n = 1 participant in the zampilimab 2000 mg group was excluded from the PK‐per protocol set due to an incorrect route of administration; an independent investigation indicated that the PK profile was suggestive of SC rather than IV administration.

#### Anti‐Drug Antibodies

Using the newly validated ADA assay, no ADAs were observed at baseline, and the incidence of ADAs was lower (occurring in one participant receiving zampilimab 2000 mg) than in the UP0029 study.

## Discussion

In healthy participants, no safety concerns were observed with zampilimab administered at an optimized infusion rate of 25 mg/min (300 mL over 120 min). Zampilimab TO was observed in human skin following administration, demonstrating a dose‐response relationship. TO increased with zampilimab dose and was directly correlated with serum zampilimab concentrations.

Zampilimab, a humanized IgG4P mAb, has evidenced the ability to inhibit extracellular TG2 activity. Additionally, in a model system where TGFβ‐1 was used to drive accumulation of ECM proteins, zampilimab effectively reduced ECM accumulation in vitro and in vivo, and the development of fibrosis in a cynomolgus monkey UUO model.^29^ The development of an immunofluorescence‐based assay in human skin biopsies demonstrated that the skin can be used as a surrogate organ to assess the TO of zampilimab.^43^ The available preclinical data supported initiation of the first‐in‐human study, UP0029, followed by the subsequent UP0105 study, which aimed to evaluate the safety, tolerability, PK, and pharmacodynamics of zampilimab administered as single IV or SC doses in healthy participants.

Due to its mechanism of action (inhibiting soluble extracellular TG2 with no intervention of the fragment crystallizable [Fc] part of the molecule), zampilimab is considered a low‐risk immunogenicity molecule, with low likelihood of cytokine reactions and severe hypersensitivity reactions.^47^, ^48^ In fact, IgG4s are commonly regarded as antibody isotypes with low Fc effector function, and therefore, low potential to induce complement derived activity and antibody dependent cellular cytotoxicity by interacting with complement of Fc gamma receptors located on immune cells.^47^, ^48^

In the Phase 1 study UP0029, no safety concerns were identified with zampilimab after single IV or SC doses up to 1000 mg. One of the five participants in the zampilimab 2000 mg IV cohort experienced an infusion‐related serious TEAE that met a pre‐defined stopping criterion, and the study was halted temporarily. However, subsequent PK modeling data for the UP0029 study suggested that doses greater than 2000 mg should be tested in order to achieve maximal TG2 occupancy at the trough concentration.

The infusion‐related reaction likely resulted from the high infusion rate and concentration of zampilimab (100 mg/mL over 60 min [33 mg/min]). Consequently, the infusion protocol was modified (lower concentration and slower rate of infusion) for the two IV doses tested in the UP0105 study, with the aim of reducing the risk of infusion reactions. In healthy participants, single IV doses of zampilimab (2000 and 3000 mg, administered over 2 h with a maximum concentration of zampilimab 10 mg/mL and maximum rate of infusion of 25 mg/min) showed a favorable safety profile with no infusion reactions reported, and no new safety signals were identified. This appears consistent with findings for other mAbs used at high doses, such as rituximab; where a slow initial infusion rate is recommended to reduce infusion reactions, which are well‐known to occur following administration of mAbs.^49^ Similarly, the mAbs daratumumab, atezolizumab, and the humanized mAb ocrelizumab, are given at lower doses during the initial infusion, and slower infusion rates are recommended until the safety profile has been established.^39^, ^40^, ^50^, ^51^

In the UP0029 study, zampilimab demonstrated linear and approximately dose‐proportional PK following single IV and SC doses up to 2000 mg. While the zampilimab serum concentration with 2000 mg dosing was consistent between the two studies, there was a slightly less than proportional increase in exposure with 3000 mg in the UP0105 study. However, the number of participants on active treatment at each dose across both studies was low (n *≤ *6), and the typical half‐life ranged from 17‐23 days across the 1000‐3000 mg dose range. This indicates that any departure from dose proportionality is likely to be small, may partly reflect methodological differences between the assays in each study, and will be explored in further studies. The estimated value for the volume distribution following IV dosing (<7 L) was similar to the predicted total blood volume, suggesting that the distribution of zampilimab was restricted to the extravascular compartment, with limited distribution to peripheral tissues, as expected for a biological agent.

Treatment with zampilimab resulted in a time‐dependent reduction in co‐localization of anti‐TG2 antibody staining, indicating that zampilimab had bound to TG2 in the skin blood vessels. A direct relationship between the serum concentration of zampilimab and TO in the skin was identified, with maximal TO at serum concentrations of approximately 250 µg/mL or higher; these results are consistent with those reported for the cynomolgus UUO preclinical model and human skin biopsy study TO/TE data.^43^

For the skin biopsies in the UP0029 study, immunofluorescence analysis showed that the most discrete expression of TG2 was in blood vessels associated with neovascularization; this is consistent with data from a rat model that showed TG activity at wound sites of neovascularization and granulation tissue formation.^46^

As with any therapeutic protein, the use of zampilimab may lead to the development of ADAs; however, the characterization of ADAs to zampilimab and their clinical consequences could not be determined, due to the limited data that are available in our studies and the duration of exposure (limited to a single dose). Although the prevalence of pre‐ADAs to human antibodies, such as zampilimab, is generally expected to be low, a rather high percentage of pre‐dose ADAs (26%) were detected in the UP0029 study. Additionally, in this study, the generation of ADAs following zampilimab dosing (IV or SC) was observed in 20 of 55 (36.4%) participants. However, these high percentages are believed to be due to the use of a suboptimal ADA assay associated with a high FPR. Consequently, a new validated ADA method with good sensitivity, balanced with appropriate drug tolerance and an improved statistical CP determination strategy, was applied (using an iterative mixed‐effects model and statistical analysis considering the distribution of the data) in the UP0105 study, thus resulting in optimal FPRs (i.e., low treatment‐emergent ADA incidence was observed in the UP0105 study compared with UP0029). The use of different ADA assays in both studies may have differentially affected the observed ADA results and may explain the inconsistency in the observed ADA results between the two studies.

Following the results of the UP0029 study, a Phase 2 study (NCT04335578) assessing the long‐term safety, tolerability, PK, and pharmacodynamics of zampilimab in adult kidney transplant recipients with chronic allograft injury and post‐renal transplant fibrosis was initiated in 2019 but was terminated in December 2021 (global end of trial May 2022) due to recruitment challenges.

## Conclusion

In conclusion, no safety concerns were identified with zampilimab at single ascending doses up to 1000 mg IV or SC and at higher IV single doses of 2000 and 3000 mg, with an optimized concentration (10 mg/mL) and infusion rate of 25 mg/min (300 mL over 120 min), in healthy participants. To date, the results of preclinical and Phase 1 studies support the continued clinical development of zampilimab for the potential treatment of diseases characterized by ECM accumulation leading to fibrosis.

## Conflicts of Interest

J.C., R.B., R.N., M.S., and Z.A. are employees of UCB and may hold/have access to shares and/or stock options. Z.A. holds/has access to GSK and Pfizer stock options. G.I.J., J.Y.C.C., T.S.S., and E.T. were employees of UCB at the time the work was completed and may have held/had access to stock options during this time. A.B. was an employee of OracleBio, and A.R. was an employee of Veramed at the time the study was conducted. UCB developed zampilimab and contracted the services of OracleBio and Veramed. A.R. is an employee of F. Hoffmann‐La Roche and holds/has access to stock options.

## Funding

The UP0029 and UP0105 studies were funded by UCB.

## Supporting information



Supporting Information

## Data Availability

Due to the small sample size in this trial, individual patient‐level data cannot be adequately anonymized as there is a reasonable likelihood that individual participants could be re‐identified. For this reason, data from this trial cannot be shared.
