# Supplementary material for: Randomized Phase 1 Studies Evaluating the Safety, Tolerability, Pharmacokinetics, and Target Occupancy of Zampilimab in Healthy Participants
Source: Clin Pharmacol Drug Dev. 2026 May 13;15:e70052. doi: 10.1002/cpdd.70052 (PMC13170637; doi:10.1002/cpdd.70052)
Supplement: Supplementary file 1 — Supporting Information [file CPDD-15-0-s001.docx]

**Supplemental Information
Randomized Phase 1 Studies Evaluating the Safety, Tolerability, Pharmacokinetics, and Target Occupancy of Zampilimab in Healthy Participants**

**Jo Collier^1^, Rowann Bowcutt^1^, Geoffrey I. Johnston^1,a^, Jane Y.C. Chan^1,b^,
Anastasiia Raievska^2,c^, Alison Bigley^3,a^, Richard Nicholl^1^, Tim S. Schmidt^1,d^,
Maria Sarno^1^, Zahid Ali^1^, Elizabeth Thomson^1,e^**

^1^UCB, Slough, UK

^2^Veramed, London, UK

^3^OracleBio, Biocity, Scotland, UK

**Current Affiliations:**

^a^These authors have since retired from their places of employment

^b^AviadoBio, London, UK

^c^F. Hoffmann-La Roche, Basel, Switzerland

^d^Senisca Ltd., Exeter, UK

^e^Elizabeth Thomson Limited, London, UK

**Corresponding Author:**

Jo Collier, MBChB, UCB, 216 Bath Road, Slough, SL1 3WE, UK

(e-mail: [Jo.Collier@ucb.com](mailto:Jo.Collier@ucb.com); phone: +44 1 753 534 655)

**Funding:**

The UP0029 and UP0105 studies were funded by UCB.

**Table S1.** Participant Key Inclusion and Exclusion Criteria for the UP0029 and
UP0105 Studies

| Inclusion criteria |
| --- |
| - Males or females between 18 and 55 years of age, at screening |
| - Adequate venous access on inspection |
| - Clinical laboratory test results within the reference ranges of the testing laboratory or outside the reference range of the laboratory but considered as not clinically significant by the Investigator |
| - Normal weight as determined by a body mass index of between 18.0 and 32.0 kg/m^2^ (with a body weight of 50–90 kg) |
| - In good physical and mental health, in the opinion of the Investigator, determined on the basis of medical history and general clinical examination at screening and at baseline |
| - ECG was considered normal or abnormal but clinically nonsignificant (as interpreted by  the Investigator) |
| Exclusion criteria |
| - Had participated in another study of an investigational medical product (or a medical device under investigation) <3 months or 5 half-lives prior to screening, whichever  was longer |
| - A known hypersensitivity to any components of zampilimab/placebo |
| - Had a history of moderate to severe allergic reaction to medication(s), including biologics^a^ |
| - Tested positive for HIV-1 or ‑2 Ab, hepatitis B virus surface antigen, or hepatitis C virus Ab  at screening |
| - Had received any prescription or non-prescription medicines (other than vitamins within the recommended daily dose limits), within 14 days or 5 half-lives of the respective drug, whichever was longer, other than the occasional use of analgesics, such as paracetamol or ibuprofen, oral contraceptives, or inhaled corticosteroids for seasonal rhinitis |
| - Smoked >10 cigarettes/day or equivalent, and was unable or unwilling to stop smoking for the period of the study |
| - Had excessive caffeinated beverage use up to 72 hours prior to baseline (a consumption of >300 mg of caffeine/day) |
| - Had over the upper limit of normal for any of the following: ALT, AST, alkaline phosphatase, or total bilirubin |
| - Had any of the following hematological function tests results at screening:  hemoglobin: <111 g/L (for women) or <113 g/L (for men); absolute neutrophil count  <1.5 x 10^9^ L (<1500 mm^3^); platelets <150 x 10^9^ L |
| - An active infection at screening |
| - Had excessive physical exertion up to 72 hours prior to baseline |
| - A known history of celiac disease |
| - Female participants who were breastfeeding, pregnant, or planned to become pregnant during the study or <6 months following the final dose |

Ab, antibody; ALT, alanine aminotransferase; AST, aspartate aminotransferase;
ECG, electrocardiogram; HIV, human immunodeficiency virus.

^a^Participants in Cohort 11 of UP0029 and participants in UP0105.

**Table S2.** Blood Sampling for Zampilimab Serum Concentration in the UP0029 Study

| Cohort | Time after start of IV infusion/SC administration |
| --- | --- |
| 1–6 | Predose, immediately after the end of infusion/administration, 2, 8, 24, 48, 72, 96, 168 (Day 8), and 336 hours (Day 15) after infusion/administration |
| 7–9^a^ | Predose, immediately after the end of infusion/administration, 8, 24, 72, 96, 168 (Day 8), 336 (Day 15), 504 (Day 22), and  1008 hours (Day 43) after infusion/administration |
| 11 | Predose, immediately after the end of infusion/administration, 8, 24, 72, 96, 168 (Day 8), 336 (Day 15), 504 (Day 22), 1008 hours (Day 43), 1704 (Day 72), and 2856 hours (Day 120) after infusion/administration |

IV, intravenous; SC, subcutaneous; TEAE, treatment-emergent adverse event.

^a^As a result of a serious TEAE suggestive of infusion reaction in Cohort 9, the study was temporarily halted, and Cohort 10 was
not enrolled.

| n (%) | Zampilimab  IV administration | | | | | | | Zampilimab SC administration | | | |  | |  |  |
| --- | --- | --- | --- | --- | --- | --- | --- | --- | --- | --- | --- | --- | --- | --- | --- |
|  | Cohort  1 10 mg  n = 6 | Cohort  2 50 mg  n = 6 | Cohort  4 250 mg  n = 6 | Cohort  6 500 mg  n = 6 | Cohort  8 1000 mg  n = 6 | Cohort  9 2000 mg  n = 5 | Cohort  3 250 mg  n = 6 | | Cohorts 5/11^b^ 500 mg  n = 11 | Cohort  7 1000 mg  n = 6 | PBO  total  n = 20 | | All participants  N = 78 | | |
| Any previous and ongoing medical conditions | 4 (66.7) | 2 (33.3) | 2 (33.3) | 4 (66.7) | 2 (33.3) | 3 (60) | 4 (66.7) | | 6 (54.5) | 3 (50) | 11 (55) | | 41 (52.6) | | |
| Seasonal allergy | 2 (33.3) | 1(16.7) | 1 (16.7) | 0 | 0 | 1 (20) | 0 | | 0 | 0 | 1 (5) | | 6 (7.7) | | |
| Rhinitis allergic | 0 | 1 (16.7) | 0 | 0 | 0 | 0 | 2 (33.3) | | 0 | 0 | 1 (5) | | 4 (5.1) | | |
| Adjustment disorder with depressed mood | 0 | 0 | 1 (16.7) | 1 (16.7) | 0 | 0 | 0 | | 1 (9.1) | 2 (33.3) | 0 | | 5 (6.4) | | |
| Any prior medication | 0 | 0 | 0 | 0 | 6 (100) | 5 (100) | 0 | | 5 (45.5) | 6 (100) | 8 (40) | | 30 (38.5) | | |
| Mupirocin^a^ | 0 | 0 | 0 | 0 | 6 (100) | 5 (100) | 0 | | 5 (45.5) | 6 (100) | 8 (40) | | 30 (38.5) | | |
| Lidocaine^a^ | 0 | 0 | 0 | 0 | 6 (100) | 0 | 0 | | 0 | 6 (100) | 4 (20) | | 16 (20.5) | | |
| Xylocaine-epinephrine^a^ | 0 | 0 | 0 | 0 | 0 | 5 (100) | 0 | | 5 (45.5) | 0 | 4 (20) | | 14 (17.9) | | |
| Any concomitant medication | 0 | 2 (33.3) | 2 (33.3) | 3 (50) | 6 (100) | 5 (100) | 1 (16.7) | | 7 (63.6) | 6 (100) | 14 (70) | | 46 (59) | | |
| Mupirocin^a^ | 0 | 0 | 0 | 0 | 6 (100) | 5 (100) | 0 | | 5 (45.5) | 5 (83.3) | 8 (40) | | 29 (37.2) | | |
| Lidocaine^a^ | 0 | 0 | 0 | 0 | 6 (100) | 0 | 0 | | 1 (9.1) | 6 (100) | 4 (20) | | 17 (21.8) | | |
| Xylocaine-epinephrine^a^ | 0 | 0 | 0 | 0 | 0 | 5 (100) | 0 | | 5 (45.5) | 0 | 4 (20) | | 14 (17.9) | | |
| Paracetamol | 0 | 0 | 2 (33.3) | 2 (33.3) | 4 (66.7) | 1 (20) | 0 | | 2 (18.2) | 1 (16.7) | 10 (50) | | 22 (28.2) | | |

**Table S3.** Previous and Ongoing Medical Conditions, Prior and Concomitant Medication in ≥2 Participants in Any Treatment Group in the UP0029 Study (FAS)

FAS, full analysis set; IV, intravenous; PBO, placebo; SC, subcutaneous; SMC, Safety Monitoring Committee.

^a^Mupirocin, lidocaine, and xylocaine-epinephrine were used with the skin biopsy sample collection in this study.

^b^Cohort 11 was added following the study hold, as the SMC recommended a repeat SC dose of 500 mg.

**Table S4.** TEAEs and Drug-Related TEAEs by MedDRA Preferred Term Reported by ≥2 Participants in Any Treatment Group in the
UP0029 Study (FAS)

| n (%), [#] |  | | Zampilimab  IV administration | | | | | | |  | | | Zampilimab  SC administration | | | | | | |  | | |  | |
| --- | --- | --- | --- | --- | --- | --- | --- | --- | --- | --- | --- | --- | --- | --- | --- | --- | --- | --- | --- | --- | --- | --- | --- | --- |
|  | PBO  IV  n = 12 | Cohort 1  10 mg  n = 6 | | Cohort 2 50 mg  n = 6 | Cohort 4 250 mg  n = 6 | Cohort 6 500 mg  n = 6 | Cohort  8 1000 mg  n = 6_a_ | Cohort  9 2000 mg  n = 5 | | | PBO  SC  n = 8 | | | Cohort 3 250 mg  n = 6 | | Cohort 5/11^b^ 500 mg  n = 11 | | Cohort  7 1000 mg  n = 6 | | | PBO  total  n = 20 | Zampilimab total  n = 58 | |  |
| Any TEAE | 5 (41.7) [11] | 2 (33.3) [2] | | 1 (16.7) [1] | 1 (16.7) [1] | 2 (33.3) [2] | 5 (83.3) [10] | 2 (40)  [2] | | | 4 (50) [5] | | | 3 (50) [5] | | 4 (36.4) [6] | | 4 (66.7)  [4] | | | 9 (45) [16] | 24 (41.4)  [33] | |  |
| Nasopharyngitis | 5 (41.7) [5] | 2 (33.3) [2] | | 1 (16.7) [1] | 0 | 2 (33.3) [2] | 3 (50)  [4] | 0 | | | 1 (12.5) [1] | | | 2 (33.3) [2] | | 2 (18.2) [2] | | 0 | | | 6 (30)  [6] | 12 (20.7)  [13] | |  |
| Headache | 2 (16.7) [3] | 0 | | 0 | 0 | 0 | 3 (50)  [3] | 0 | | | 2 (25) [3] | | | 0 | | 2 (18.2) [3] | | 1 (16.7) [1] | | | 4 (20)  [6] | 6 (10.3)  [7] | |  |
| Influenza-like illness | 0 | 0 | | 0 | 1 (16.7) [1] | 0 | 0 | 0 | | | 0 | | | 2 (33.3) [3] | | 0 | | 1 (16.7)  [1] | | | 0 | 4 (6.9)  [5] | |  |
| Procedural pain | 1 (8.3) [3] | 0 | | 0 | 0 | 0 | 1 (16.7) [1] | 2 (40)  [2] | | | 0 | | | 0 | | 1 (9.1)  [1] | | 0 | | | 1 (5)  [3] | 4 (6.9)  [4] | |  |
| Incision site pruritus | 0 | 0 | | 0 | 0 | 0 | 2 (33.3) [2] | 0 | | | 1 (12.5) [1] | | | 0 | | 0 | | 0 | | | 1 (5)  [1] | 2 (3.4)  [2] | |  |
| Hot flush | 0 | 0 | | 0 | 0 | 0 | 0 | 0 | | | 0 | | | 0 | | 0 | | 2 (33.3) [2] | | | 0 | 2 (3.4)  [2] | |  |
| Drug-related TEAEs |  |  | |  |  |  |  |  | | |  | | |  | |  | |  | | |  |  | |  |
| Any related TEAE | 3 (25) [6] | 0 | | 1 (16.7) [1] | 2 (33.3) [2] | 1 (16.7) [1] | 3 (50)  [4] | 2 (40)  [2] | | | 0 | | | 1 (16.7) [1] | | 2 (18.2) [4] | | 4 (66.7) [8] | | | 3 (15)  [6] | 16 (27.6)  [23] | |  |
| Infusion-related reaction | 0 | 0 | | 0 | 0 | 1 (16.7) [1] | 0 | 1 (20)  [1] | | | 0 | | | 0 | | 0 | | 0 | | | 0 | 2 (3.4)  [2] | |  |
| Headache | 2 (16.7) [2] | 0 | | 0 | 0 | 0 | 2 (33.3) [2] | 0 | | | 0 | | | 0 | | 1 (9.1) [2] | | 1 (16.7) [1] | | | 2 (10)  [2] | 4 (6.9)  [5] | |  |
| Nasal congestion | 0 | 0 | | 0 | 0 | 0 | 1 (16.7) [1] | 0 | 0 | | | 0 | | | 0 | | 1 (16.7) [1] | | 0 | | | 2 (3.4)  [2] | |  |
| Hot flush | 0 | 0 | | 0 | 0 | 0 | 0 | 0 | 0 | | | 0 | | | 0 | | 2 (3.3)  [2] | | 0 | | | 2 (3.4)  [2] | |  |

TEAEs were defined as any event that was not present prior to the administration of zampilimab/PBO, or any unresolved event present before administration that worsened in intensity following exposure to the treatment; TEAEs were coded using MedDRA v20.1. Drug-related TEAEs were defined as those with a relationship of 'related' or those with missing relationship.

FAS, full analysis set; IV, intravenous; MedDRA, Medical Dictionary for Regulatory Activities; PBO, placebo; SC, subcutaneous;
SMC, Safety Monitoring Committee; TEAE, treatment-emergent adverse event.

^a^One participant from this group withdrew from the study. No TEAE data were collected for this participant after Day 15.

^b^Cohort 11 was added following the study hold, as the SMC recommended a repeat SC dose of 500 mg.

[#] is the individual occurrences of the defined TEAE.

**Table S5.** Blood Sampling for Zampilimab Serum Concentration in the UP0105 Study

| Cohort | Time after start of IV infusion |
| --- | --- |
| 2000 mg and 3000 mg | Predose, within 10 minutes after the end of infusion, 4, 8, 24, 48, 72, 96, 168 (Day 8), 336 (Day 15), 504  (Day 22), 672 (Day 29), 1008 (Day 43), 1704 (Day 72), and 2856 hours (Day 120) after the start of the infusion, and at the withdrawal visit |

IV, intravenous.

**Table S6.** Previous and Ongoing Medical Conditions, Prior and Concomitant Medication in the UP0105 Study (SAS)

| n (%) | Zampilimab IV  2000 mg  n = 6 | Zampilimab IV  3000 mg  n = 6 | PBO  total  n = 4 | All participants  N = 16 |
| --- | --- | --- | --- | --- |
| Any previous and ongoing medical conditions | 5 (83.3) | 3 (50) | 3 (75) | 11 (68.8) |
| Amblyopia | 1 (16.7) | 0 | 0 | 1 (6.3) |
| Seasonal allergy | 0 | 1 (16.7) | 3 (75) | 4 (25) |
| Appendicitis | 1 (16.7) | 0 | 0 | 1 (6.3) |
| Femur fracture | 1 (16.7) | 0 | 0 | 1 (6.3) |
| Arthralgia | 0 | 1 (16.7) | 0 | 1 (6.3) |
| Adjustment disorder with depressed mood | 0 | 1 (16.7) | 0 | 1 (6.3) |
| Post-traumatic stress disorder | 1 (16.7) | 0 | 0 | 1 (6.3) |
| Rhinitis allergic | 1 (16.7) | 0 | 0 | 1 (6.3) |
| Eczema | 1 (16.7) | 0 | 0 | 1 (6.3) |
| Any prior medication | 3 (50) | 3 (50) | 3 (75) | 9 (56.3) |
| Vitamins^a^ | 2 (33.3) | 0 | 2 (50) | 4 (25) |
| Hormonal contraceptive^b^ | 1 (16.7) | 1 (16.7) | 1 (25) | 3 (18.8) |
| Paracetamol | 0 | 3 (50) | 0 | 3 (18.8) |
| Any concomitant medication | 2 (33.3) | 3 (50) | 1 (25) | 6 (37.5) |
| Tetanus vaccine | 0 | 1 (16.7) | 0 | 1 (6.3) |
| Hormonal contraceptives^b^ | 1 (16.7) | 1 (16.7) | 1 (25) | 3 (18.8) |
| Naproxen | 0 | 1 (16.7) | 0 | 1 (6.3) |
| Paracetamol | 2 (33.3) | 2 (33.3) | 0 | 4 (25) |
| Dihydrocodeine bitartrate; paracetamol | 0 | 1 (16.7) | 0 | 1 (6.3) |

IV, intravenous; PBO, placebo; SAS, safety analysis set.

^a^Includes: Vitamin C, multivitamins, Vitamin A and D (including with cod-liver oil), Vitamin B complex (including combinations with Vitamin C, biotin, calcium carbonate, and magnesium).

^b^Includes: drospirenone;ethinylestradiol, ethinylestradiol;levonorgestrel, ethinylestradiol;norgestimate.

**Table S7.** TEAEs by MedDRA Preferred Term Reported by ≥1 Participants in Any Treatment Group in the UP0105 Study (SAS)

| n (%), [#] | PBO  total  n = 4 | Zampilimab IV  2000 mg  n = 6 | Zampilimab IV  3000 mg  n = 6 | Zampilimab  total  n = 12 |
| --- | --- | --- | --- | --- |
| Any TEAE | (25) [1] | 3 (50) [4] | 5 (83.3) [14] | 8 (66.7) [18] |
| Pyrexia | 0 | 0 | 1 (16.7) [1] | 1 (8.3) [1] |
| Catheter site bruise | 1 (25) [1] | 0 | 0 | 0 |
| Contusion | 0 | 0 | 1 (16.7) [1] | 1 (8.3) [1] |
| Skin laceration | 0 | 0 | 1 (16.7) [1] | 1 (8.3) [1] |
| Aspartate aminotransferase increased | 0 | 0 | 2 (33.3) [2] | 2 (16.7) [2] |
| Blood creatine phosphokinase increased | 0 | 0 | 2 (33.3) [2] | 2 (16.7) [2] |
| Blood lactate dehydrogenase increased | 0 | 0 | 2 (33.3) [2] | 2 (16.7) [2] |
| Back pain | 0 | 0 | 1 (16.7) [1] | 1 (8.3) [1] |
| Musculoskeletal pain | 0 | 0 | 1 (16.7) [1] | 1 (8.3) [1] |
| Headache | 0 | 2 (33.3) [2] | 1 (16.7) [1] | 3 (25) [3] |
| Epistaxis | 0 | 0 | 1 (16.7) [1] | 1 (8.3) [1] |
| Oropharyngeal pain | 0 | 0 | 1 (16.7) [1] | 1 (8.3) [1] |
| Dermatitis contact | 0 | 1 (16.7) [2] | 0 | 1 (8.3) [1] |

TEAEs were defined as any event that was not present prior to the administration of zampilimab/PBO, or any unresolved event present before administration that worsened in intensity following exposure to the treatment; TEAEs were coded using MedDRA v20.1.

IV, intravenous; MedDRA, Medical Dictionary for Regulatory Activities; PBO, placebo; SAS, safety analysis set; TEAE, treatment-emergent adverse event.

[#] is the individual occurrences of the defined TEAE.

**
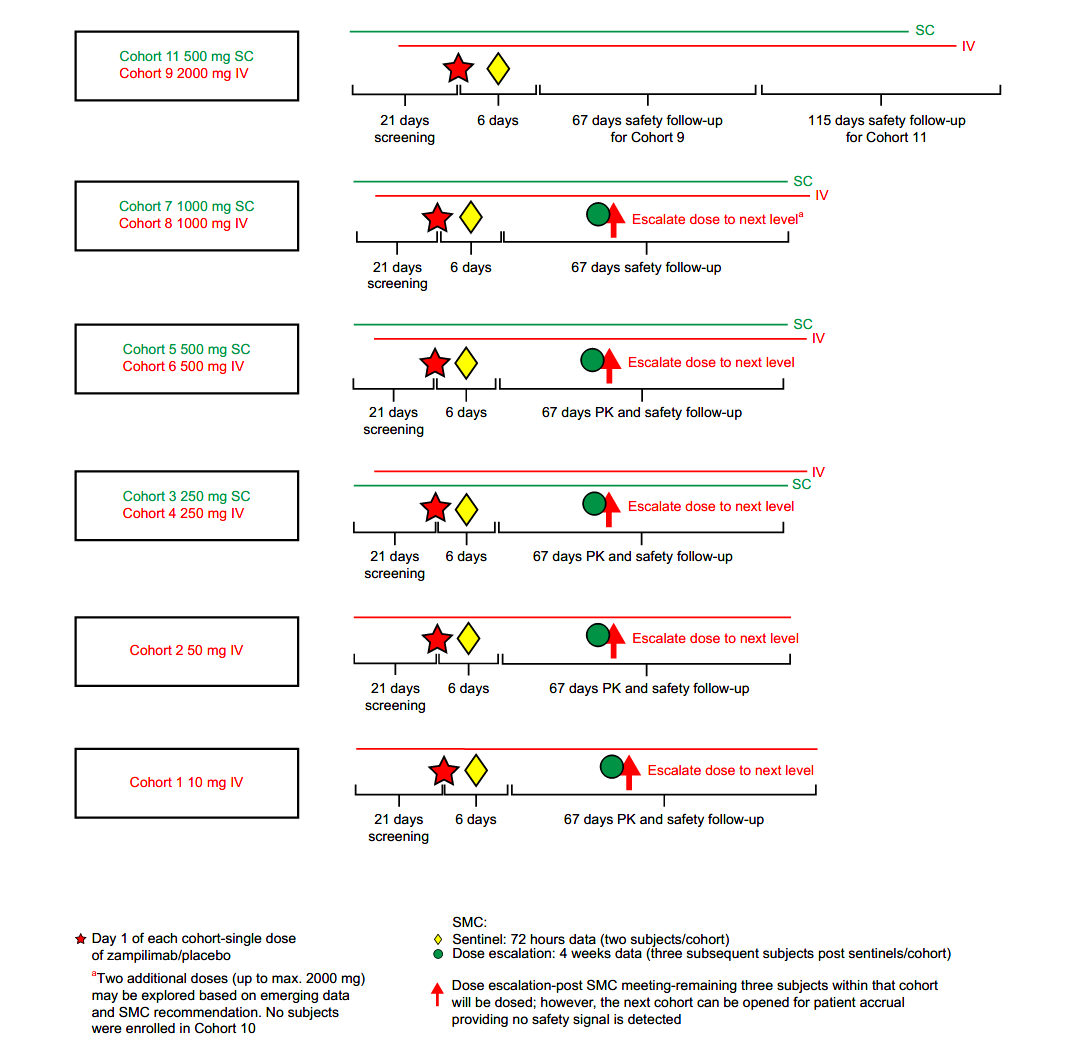
**

**Figure S1.** Study design for UP0029.

IV, intravenous; max, maximum; PK, pharmacokinetics; SC, subcutaneous; SMC, Safety Monitoring Committee.


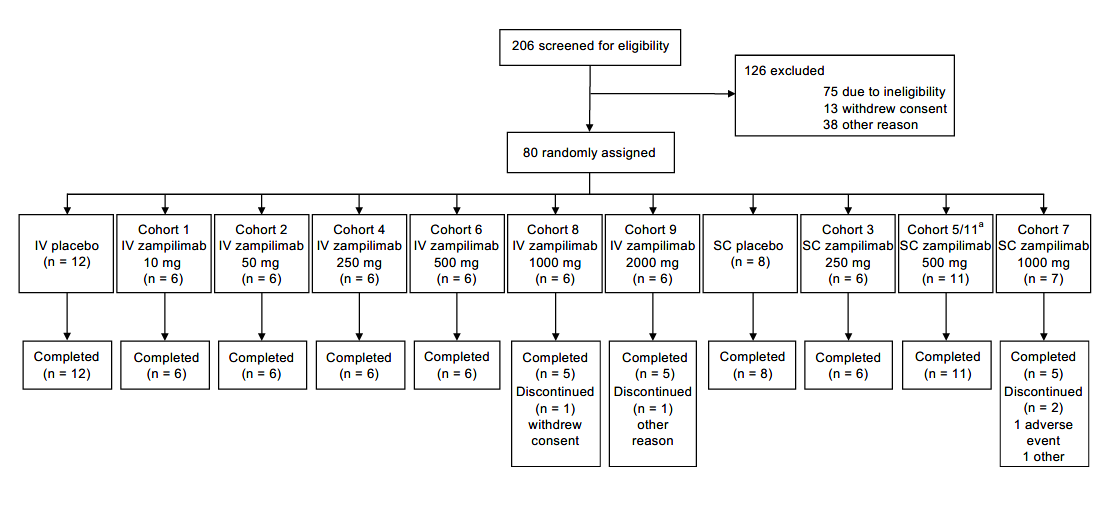


**Figure S2.** UP0029 Phase 1 first-in-human study CONSORT diagram of participant disposition.

IV, intravenous; SC, subcutaneous; SMC, Safety Monitoring Committee. ^a^Cohort 11 was added following the study hold, as the SMC recommended a repeat SC dose of 500 mg.

**
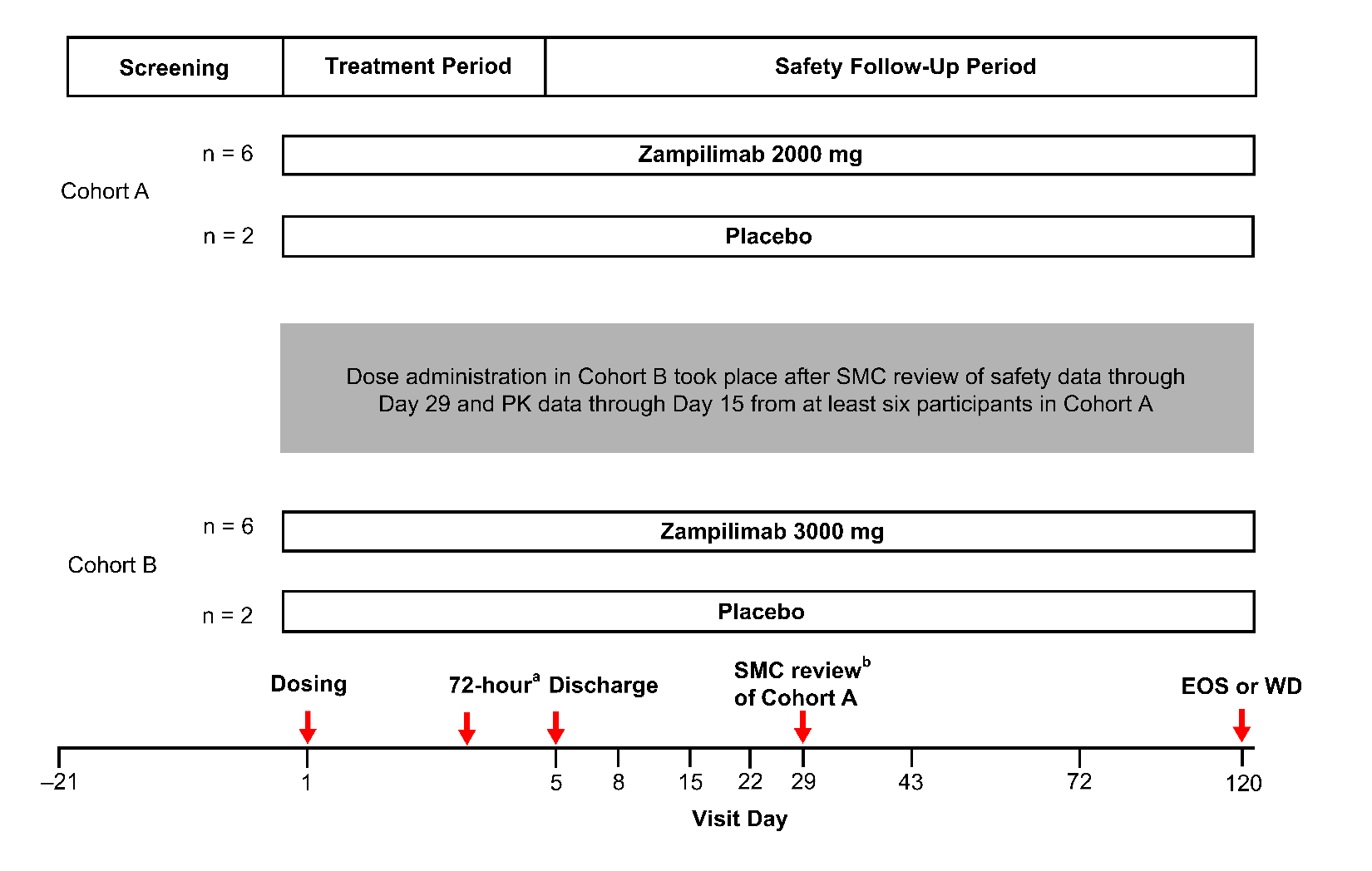
****Figure S3.** Study design for UP0105.

EOS, end of study; PK, pharmacokinetics; SMC, Safety Monitoring Committee; WD, withdrawal.

^a^Safety was confirmed by the SMC for the first two study participants of each cohort (72-hour observation period) before subsequent participants within that cohort received zampilimab.

^b^The SMC reviewed safety data (up to Day 29) and PK data (up to Day 15) from at least six participants in Cohort A to determine whether escalation to Cohort B could proceed.

**
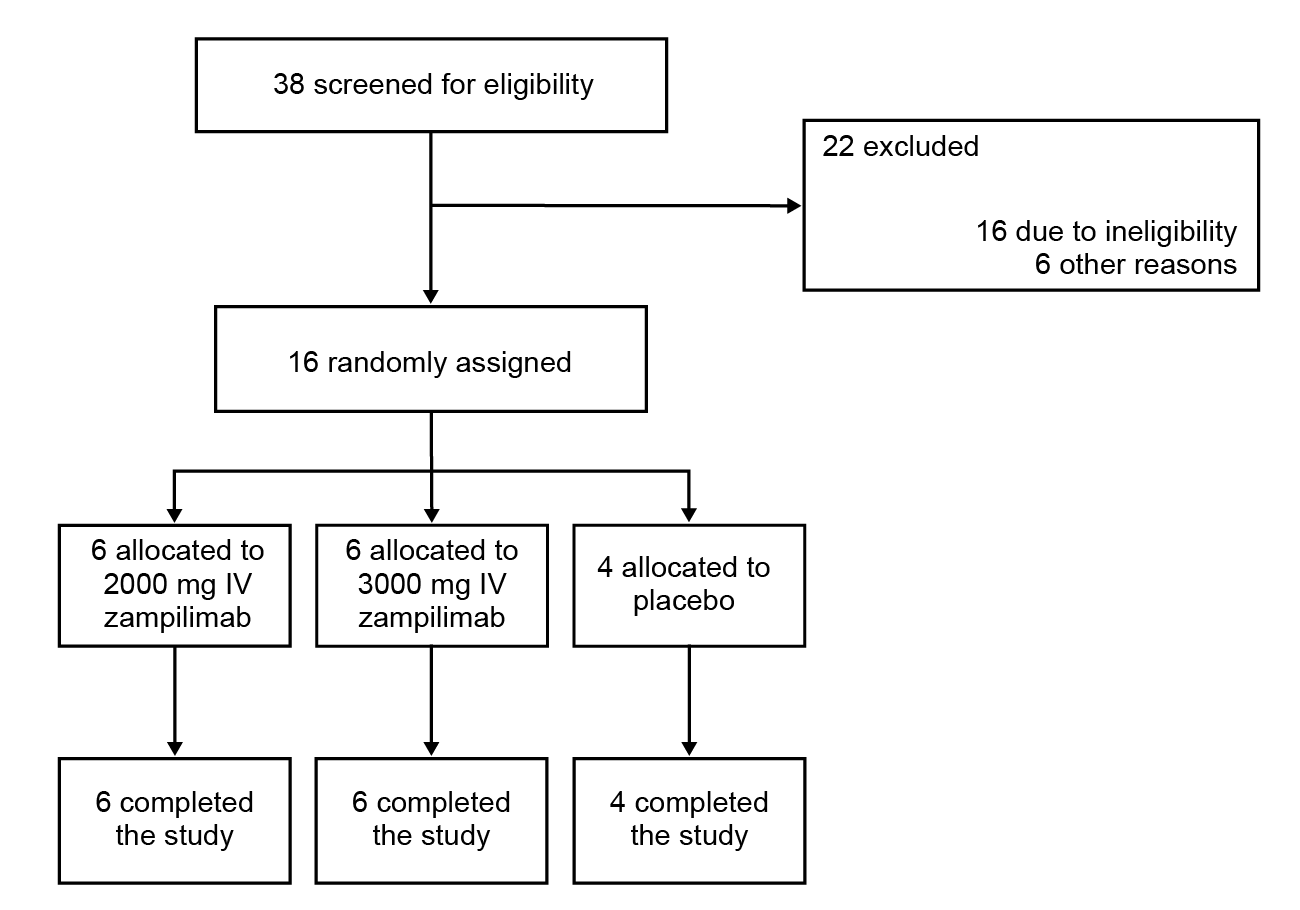
**

**Figure S4.** UP0105 Phase 1 safety study CONSORT diagram of participant disposition.

IV, intravenous
